# The global distribution of *Plasmodium vivax* and G6PD deficiency prevalence

**DOI:** 10.1186/1475-2875-11-S1-O2

**Published:** 2012-10-15

**Authors:** Simon I Hay

**Affiliations:** 1 University of Oxford, UK

## 

Existing understanding of the spatial epidemiology and geographical distribution of *Plasmodium vivax* is poor. Here we present the first systematic effort to map its endemicity globally. Routine case reporting data were assembled from 17,893 administrative units across the 95 *P. vivax* endemic countries and combined with biological risk exclusion layers and other medical intelligence data to update the estimated global limits of *P. vivax* transmission for 2010. Within areas of stable transmission, a second assembly of 9,970 quality-checked and geopositioned *P. vivax* parasite rate surveys were used with a spatiotemporal Bayesian model-based geostatistical approach to estimate endemicity age-standardised to the 1-99 year age range within every 5×5 km resolution grid square. The model incorporated prevalence data on the refractory Duffy negativity phenotype to appropriately suppress risk predictions, particularly in Africa. Endemicity was predicted within a relatively narrow range of prevalence throughout the endemic world with the point estimate rarely exceeding 7%. These patterns are described. Radical cure of *P. vivax* requires treatment of the parasite’s dormant relapsing life stages, for which primaquine is the only drug licenced. This drug may, however, trigger mild to severe haemolysis in patients with a genetically determined deficiency in glucose-6-phosphate dehydrogenase production (G6PDd). We therefore also present the first evidence-based continuous prevalence map of G6PDd globally. Representative community surveys of phenotypic G6PDd prevalence were identified for 1,734 spatially-unique sites globally. These formed the evidence-base for a Bayesian geostatistical model adapted to the G6PD gene’s X-linked inheritance mechanism, which generated a G6PDd allele frequency map across malaria endemic countries. The resulting maps and population estimates reflect potential risk of primaquine-associated harm.

